# Harnessing DNA computing and nanopore decoding for practical applications: from informatics to microRNA-targeting diagnostics

**DOI:** 10.1039/d3cs00396e

**Published:** 2024-10-29

**Authors:** Sotaro Takiguchi, Nanami Takeuchi, Vasily Shenshin, Guillaume Gines, Anthony J. Genot, Jeff Nivala, Yannick Rondelez, Ryuji Kawano

**Affiliations:** a Department of Biotechnology and Life Science, Tokyo University of Agriculture and Technology Koganei-shi Tokyo 184-8588 Japan rjkawano@cc.tuat.ac.jp; b Laboratoire Gulliver, CNRS, ESPCI Paris, PSL Research University 10 rue Vauquelin Paris 75005 France guillaume.gines@espci.fr yannick.rondelez@espci.fr; c LIMMS, CNRS-Institute of Industrial Science, University of Tokyo Meguro-ku Tokyo 153-8505 Japan genot@iis.u-tokyo.ac.jp; d Paul G. Allen School of Computer Science and Engineering, University of Washington Seattle WA USA jmdn@cs.washington.edu; e Molecular Engineering and Sciences Institute, University of Washington Seattle WA USA

## Abstract

DNA computing represents a subfield of molecular computing with the potential to become a significant area of next-generation computation due to the high programmability inherent in the sequence-dependent molecular behaviour of DNA. Recent studies in DNA computing have extended from mathematical informatics to biomedical applications, with a particular focus on diagnostics that exploit the biocompatibility of DNA molecules. The output of DNA computing devices is encoded in nucleic acid molecules, which must then be decoded into human-recognizable signals for practical applications. Nanopore technology, which utilizes an electrical and label-free decoding approach, provides a unique platform to bridge DNA and electronic computing for practical use. In this tutorial review, we summarise the fundamental knowledge, technologies, and methodologies of DNA computing (logic gates, circuits, neural networks, and non-DNA input circuity). We then focus on nanopore-based decoding, and highlight recent advances in medical diagnostics targeting microRNAs as biomarkers. Finally, we conclude with the potential and challenges for the practical implementation of these techniques. We hope that this tutorial will provide a comprehensive insight and enable the general reader to grasp the fundamental principles and diverse applications of DNA computing and nanopore decoding, and will inspire a wide range of scientists to explore and push the boundaries of these technologies.

## Abstract 2

This tutorial review provides fundamentals on DNA computing and nanopore-based decoding, highlighting recent advances towards microRNA-targeting diagnostic applications.

## Graphical abstract

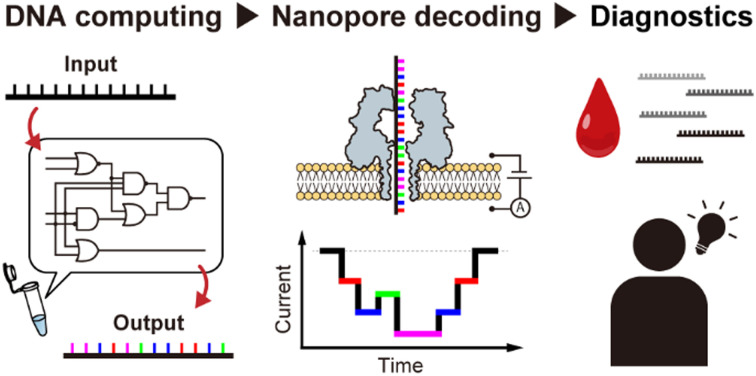

**Key learning points**

(1) Definition of DNA computing and its historical developing trajectory.

(2) Basic strategies to construct DNA-based logic gates, circuits, and neural networks, including approaches to connect chemical signals (small molecules) to DNA computing.

(3) Nanopore decoding: approaches to utilise nanopore technology as a decoder for DNA-computed output information.

(4) Strategies for expanding medical diagnostics such as microRNA-targeting diagnostics using both DNA computing and nanopore decoding.

(5) Current challenges and perspectives towards practical implementation.

## 1. Introduction

DNA, the common biomolecule of all organisms, is a polymer chain of four distinct deoxynucleotide monomers, denoted as A, T, G, and C. In living organisms, the sequence of the monomers encodes the genetic information, which is read out and utilised *via* transcription and translation. From a materials science perspective, DNA is a sequence-programmable polymer that is amenable to low-cost chemical synthesis. Combining the strategic use of predictable Watson–Crick base pairing with sophisticated DNA manipulation techniques, such as templated replication by polymerases, sequence-specific enzymatic cleavage, and toehold-mediated strand displacement (TMSD), research into using DNA as a functional nanomaterial has expanded its scope of applications. One of the most intriguing avenues of DNA computing is the idea to “process information with DNA”, which is being developed using a combination of technological, chemical, and biochemical tools.

**Fig. 1:**
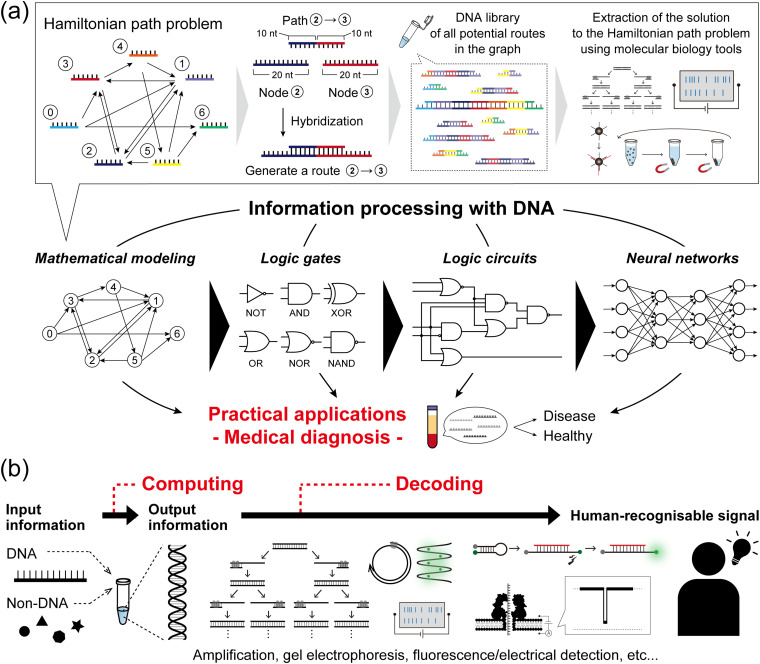
Concept of DNA computing. (a) (top) A schematic illustration of the DNA computing methodology to solve a Hamiltonian path problem. The parallel hybridization of each path strand and each node strand in the graph results in the autonomous generation of all possible routes. (bottom) The development of DNA computing from mathematical modelling to the construction of logic gates, circuits, and neural networks. These achievements have steered towards practical applications, particularly in the field of medical diagnosis. (b) In DNA computing, DNA and non-DNA molecules are exploited as input information. As important as the encoding of information, the decoding of the DNA-computed output information allows its interpretation into a human-recognisable signal *via* biochemical manipulation, fluorescence detection, or nanopore technology.

The concept of information processing with DNA molecules can be traced back to Adleman's pioneering work in 1994: computing a directed Hamiltonian path problem (HPP) using DNA.^1^ The solution to the HPP is to find a route between multiple nodes in a graph, such that each node is visited exactly once (practically known as the traveling salesman problem). One general approach to solving this type of combinatorial problem is an exhaustive search, which quickly reaches an unrealistically large number of computations as the size of the problem increases. Adleman addressed this challenge by harnessing the massive parallelism of molecular self-assembly as follows (Fig. 1a). First, each node in the graph was associated with a specific DNA strand (20-mer). Each path, connecting two nodes in the graph, was then encoded with a 20-mer DNA consisting of the partial complementary sequences (10-mer) of the two node strands. In this way, the path strands physically connect the node strands by hybridization, resulting in the creation of routes. In the case that 1 mole of DNA is present, 6.02 × 10^23^ molecules simultaneously hybridise in parallel, thereby generating a DNA assembly library encoding all potential routes within the graph. Through subsequent selection processes by biochemical manipulation, any remaining full-length DNA routes correspond to an answer to the HPP. This seminal demonstration established the concept of DNA computing and was followed by several reports on DNA-based mathematical computing.^2,3^ Originating from such mathematical modelling, the development of DNA computing next steered towards modelling digital electronics with logic gate operations, due to the inherent simplicity of handling binary information represented by ‘0’ and ‘1’. In the first report in 2002, Stojanovic *et al.* implemented NOT, AND, and XOR gates using single-stranded DNAs (ssDNAs) as inputs and deoxyribozymes (DNAzymes) as computational modules.^4^ In addition to the implementation of other basic logic gates, OR, NOR, and NAND,^5,6^ the development of TMSD^7^ (described in detail in the next section) allowed for the cascading of individual DNA logic gates and the construction of full DNA circuits.^8,9^ Departing from Boolean logic, a fascinating topic is the construction of molecular neural networks (NNs), *i.e.* DNA circuits that mimic the interconnected neuronal architecture of a brain. Following the pioneering construction of a DNA-based Hopfield network (a NN model) in 2011,^10^ several types of NN architectures have been developed such as winner-take-all NNs,^11^ convolutional NNs,^12^ and nonlinear decision-making NNs.^13^ The scale, complexity, and computational capability of DNA-based architectures have recently begun to reach the point of implementation in practical applications (Fig. 1a).

Since oligonucleotide biomarkers can inherently be used as inputs in DNA computation, many applications of this field are related to diagnostics. Among the variety of biomarkers, microRNAs (miRNAs) are promising candidates as the targets in DNA computing. In recent decades, dysregulation (up- or down-regulation) of miRNA expression has been linked to a variety of pathological disorders including cancers,^14–16^ neurodegenerative conditions,^17–19^ and cardiovascular^20–22^ or infectious diseases.^23–25^ In addition, miRNA are stably released in body fluids and can be collected by non-invasive diagnostic methods known as liquid biopsies.^26,27^ More than 2000 miRNA sequences have been identified in the human genome, suggesting that more than one miRNA ends up dysregulated during disease progression.^28,29^ This urges the development of multiplexed miRNA signature classifiers to create unique diagnostic tools.^30,31^ DNA computing allows the technical burden (and associated cost) of highly multiplex assays to be transferred to a biochemical system where miRNA transduction, information processing, and readout are integrated into a low-cost but sophisticated molecular algorithm. This concept has led to the development of molecular classifiers for miRNA pattern recognition.

As the field of DNA computing matures and expands in different directions, a critical question becomes how to connect these DNA circuits to others, such as non-nucleic acid types of molecular input. Inspired by biological principles such as allostery, the modulation of protein or enzyme function by a remote ligand interaction, researchers have expanded the range of compatible molecular-inputs, including various types of chemical signals (proteins, small molecules) through the use of transcription factors^32^ or aptamers.^33^

As important as the encoding of information, the decoding of the output of a DNA computation allows its interpretation from a molecular to a human-recognisable signal (Fig. 1b). Conventional decoding approaches, including output amplification, gel electrophoresis, and dye-labelled fluorescence detection, generally face challenges in practical implementation regarding decoding time, laborious manipulation, and equipment cost. As an alternative, nanopore technology,^34,35^ which enables the direct, label-free, single-molecule, and sequence-specific electrical detection of oligonucleotides, has emerged as an attractive decoding tool. For practical use, this nanopore-based method, called nanopore decoding,^36^ is widely accessible with the commercially available nanopore array device MinION^37^ from Oxford Nanopore Technologies, which has recently attracted attention as a multiplex DNA sequencing platform.^38,39^

In this tutorial review, we present the fundamental knowledge, technologies, and methodologies associated with (i) DNA-based construction of logic gates, circuits, and NNs and their miRNA-targeting diagnostic applications, (ii) DNA computing with non-nucleic acid inputs, (iii) nanopore decoding and its diagnostic applications, and (iv) multiplex nanopore decoding using MinION. Finally, we conclude with some prospects for their practical implementation. We hope that this review will guide general readers to understand the principles and applications of DNA computing and nanopore decoding and will help a wide range of scientists to further explore and develop these technologies.

## 2. DNA computing *via* logic gates, circuits, neural networks, and their diagnostic applications

First, we provide the fundamentals and methods for DNA-based construction of logic gates, circuits, and NNs, and then describe associated miRNA-targeting diagnostic applications that mainly use fluorescence readout.

### DNA-based logic gates and circuits

In Boolean DNA computing, binary information, represented as ‘0’ and ‘1’, is generally interpreted by the absence or presence of the sequence-designed DNAs. Here, we introduce one common strategy for constructing DNA logic gates, and two cascadable strategies for constructing DNA circuits.

**Fig. 2:**
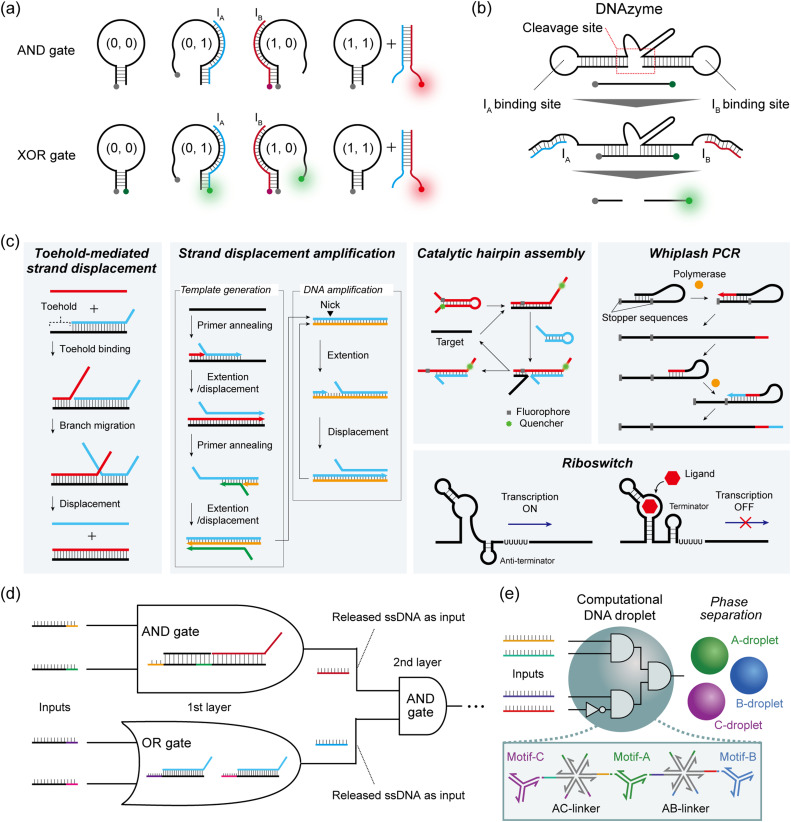
DNA-based reaction and logic gate operations. Schematic illustrations of (a) hairpin/molecular beacon-based logic gate operation (AND gate and XOR gate), (b) DNAzyme-based logic gate operation (AND gate), (c) toehold-mediated strand displacement (TMSD) reaction, strand displacement amplification (SDA), catalytic hairpin assembly (CHA), whiplash PCR, and a transcriptional riboswitch, and (d) TMSD-based DNA circuit construction. The released ssDNA in the first-layer gate is used as the input for the next-layer gate to cascade the logic gates. (e) DNA droplet made of multiple-branched DNA structures runs a Boolean algorithm. In the presence of a 4-miRNA input pattern, the DNA nanostructure including the motifs and linkers is disassembled by strand displacement, resulting in the phase separation of the orthogonal DNA droplet.

Hairpin structures or molecular beacons (MBs) are commonly used for DNA-based logic gate construction. Yang *et al.* first designed three DNA components to construct an AND gate: a hairpin DNA labelled with a quencher at the 3′ end, input I_A_ labelled with a fluorophore (Fluoro Orange) at the 5′ end, and input I_B_ without a label.^40^ I_A_ and I_B_ were designed to bind either to the hairpin or to each other. In the presence of only one of the two inputs, a weak fluorescence signal is observed, either because I_A_ is quenched upon hybridization with the hairpin or simply because I_B_ has no fluorophore (= output ‘0’). When both I_A_ and I_B_ are present, these inputs bind to each other, resulting in the emission of a fluorescence signal from the Fluoro Orange label (= output ‘1’) (Fig. 2a). To construct the XOR gate, they subsequently added another fluorophore label (FAM) at the 5′ end of the hairpin to create the quencher-labelled MB (Fig. 2a). In the case of no input, FAM fluorescence was quenched within the hairpin. By inputting either I_A_ or I_B_, the opening of the hairpin induced FAM fluorescence (green) (= output ‘1’), whereas I_A_ (Fluoro Orange) was quenched by the quencher on the MB. When both I_A_ and I_B_ were input together, the I_A_–I_B_ duplex was formed and the MB remained in the closed hairpin structure, leading to the on state of the Fluoro Orange red signal (= output ‘0’). As described here, by modulating the fluorescence output and its logical definition, various DNA-based logic gates can be customised using this hairpin/MB approach.

To cascade individual gates in the construction of DNA circuits, the first strategy used DNAzymes that selectively cleave DNA strands with metal ion cofactors. In Stojanovic's pioneering work, two ssDNAs (I_A_ and I_B_) were defined as inputs, and a DNAzyme acted as a computational module that logically generated different outputs when cleaving the DNA substrates in response to the inputs.^4^ I_A_ and I_B_ were designed to bind with the cleavage site of the DNAzyme and activate it by a binding-induced conformational change, resulting in the cleavage of the fluorescence-quenched DNA substrate only when the output is ‘1’. This molecular behaviour can be identified by an increase in fluorescence intensity (Fig. 2b). Using this design rule, they implemented NOT and AND logic gates, as well as the more complex XOR gate. Using a similar design, Orbach *et al.* constructed a DNA circuit by cascading different DNAzyme logic gates to achieve the functions of a half-adder and a full-adder for binary numbers.^41^

The second cascadable strategy uses TMSD (Fig. 2c). In the TMSD reaction, an ssDNA (input) binds to the overhanging single-stranded region (the toehold) of a DNA duplex, initiating a branch migration that displaces an existing output strand from the duplex. By incorporating a fluorescence resonance energy transfer (FRET) system in this TMSD, Seelig *et al.* constructed a two-input AND gate.^9^ In their approach, the binding of input I_A_ and the displacement of ssDNA leads to the exposure of the toehold for I_B_ binding. Subsequent I_B_ binding induces the displacement of the quenched fluorescence-labelled strands from the dsDNA. This AND gate molecular behaviour can be monitored by the increase in fluorescence intensity (= output ‘1’). By using the released ssDNA as the input for the next-layer gate, they subsequently constructed a DNA circuit that cascaded an AND gate and an OR gate (Fig. 2d). This TMSD-based cascading strategy has been widely applied to construct not only a larger DNA circuit with 130 strands^8^ but also DNA circuits with unique functions such as a temporal DNA circuit that can respond to both the presence and history of a molecular environment^42^ and a pH-responsive switchable DNA circuit.^43^

### DNA-based logic gates and circuits: towards diagnostics

#### Toehold-mediated strand displacement cascade

In the seminal work of Seelig *et al.*, the authors reported the construction of logic gates (AND, OR, and NOT) relying on TMSD reactions.^9^ The intrinsic modularity of the design allows for the assembly of intricate Boolean circuits analogous to their electronic counterparts through the cascading of multiple gates, whereby the output of one gate serves as the input of another. The larger circuit comprised 11 gates, organised into 5 layers designed to accept 6 DNA analogues of miRNA sequences. Lv *et al.* reported a multilayer DNA-based programmable gate array capable of nonlinear sample classification based on cascaded logic gates.^44^ The algorithm encodes a 3-miRNA input decision tree for classifying synthetic samples mimicking kidney renal clear cell carcinoma patients. Despite their computational capacity, making TMSD-based circuits applicable to the miRNA concentrations typically found in biological samples would necessitate their integration with either a miRNA preamplification stage or a downstream signal amplification strategy. For instance, rolling circle amplification (RCA) has been used to amplify the output of an AND gate, displaying a limit of detection (LOD) in the picomolar range.^45^ Another pitfall of chemical multilayer or cascaded circuits is the implementation of a NOT gate, which converts the absence of an input into a positive output. It is imperative that the gate remain inactive until the upstream layers have been completed; otherwise there is a significant risk of systematic and premature activation. To circumvent this potential issue, a dual-rail computational architecture has been proposed, albeit at the cost of increased circuit complexity.^8,46^ Alternatively, Emanuelson *et al.* addressed this challenge by introducing a photocaged DNA gate. In this approach, nitropiperonyloxymethylene groups are incorporated on thymidine residues in the stem of a hairpin sequence, inhibiting its natural folding. Upon exposure to UV light, the uncaged hairpin folds, expelling a positive-NOT output that can interact with downstream gates. However, if the input is present, it interacts with the loop of the hairpin, forbidding its re-folding and thereby blocking the release of the output. This photoregulation mechanism, tested on 4-miRNA input multilayer circuits, offers the flexibility to introduce a NOT gate (and associated NOR and NAND gates) at any layer. This is possible because the gate can be activated only when the upstream computation is complete, preventing premature release of the NOT output.

#### Multi-way junctions for logic gates

The implementation of more complex DNA computation is readily achieved through the utilisation of multi-way junctions, which represent a straightforward approach for encoding Boolean functions. For instance, Miao *et al.* have coupled strand displacement amplification (SDA)^47^ with a downstream DNA walker and an electrochemical readout.^48^ In this work, the target miRNA initiates a polymerase/nickase-driven SDA (Fig. 2c), whose output creates a 3-way junction (3-WJ) with the 2 single-stranded legs of the walker. As a consequence, the fully assembled bipedal walker can interact with DNA hairpins grafted on the surface of a gold electrode (the track) and mediate catalytic hairpin assembly (CHA)^49^ (Fig. 2c), with an in-solution “driver sequence” that is modified with methylene blue. As a result, the bipedal walking eventually brings this electrochemically active synthon close to the electrode, allowing miRNA sensing by square wave voltammetry. The use of multibranched DNA complexes as an SDA template allows production of the output conditionally in the presence of several miRNAs. Additionally, the logic can be reversed to generate NOT, NOR, or NAND gates, by making the SDA reaction produce an output strand that competitively inhibits formation of the walker. Shi *et al.* similarly combined a CHA process with an upstream multibranched polymerase-driven strand displacement reaction for building miRNA AND gates.^50^ Here, the CHA product, labeled with a platinum-coated gold nanoparticle, can be captured on a lateral flow test strip for a readout visible with the naked eye. The initial computing layer could be complexified by leveraging the full potential of strand-displacing polymerase.^46,51^ CHA has also been used in combination with a DNA nanotweezer to create a 2-miRNA AND gate. The design of the nanotweezer was based on a double crossover motif modified at both termini with a split DNAzyme sequence.^52^ When both miRNA targets were present, they induced the transition of the originally open tweezer to a closed conformation by sequential TMSD, subsequently leading to the formation of the active DNAzyme in the presence of hemin, which catalysed the production of a colorimetric product. Furthermore, the miRNAs were regenerated through the CHA reaction with a fuel strand. This doubly-catalytic approach (CHA + DNAzyme) endowed the assay with a LOD of 30 fM.

TMSD reactions were also exploited in conjunction with a 3-WJ to encode a 3-input miRNA AND gate with the readout based on gold nanoparticle crosslinking.^53^ Mameuda *et al.* harnessed the ability of a triple-branched DNA nanostructure to reversibly assemble a 3-enzyme cascade network – involving β-galactosidase, glucose oxidase, and horseradish peroxidase (HRP).^54^ Each branch of the structure was modified with one of the three enzymes, leading to an enhanced accumulation of the fluorescent product resorufin due to the proximity effect. In a 3-input AND gate implementation, the presence of all three targets triggered the disassembly of the structure, resulting in the reduction of fluorescence.

The Takinoue group exploited the self-assembly capability of multibranched DNA structures to form DNA droplets.^55^ Different DNA droplets can be fused by incorporating multi-arm DNA linkers, and employed to perform logical operations. Upon encountering a specific miRNA pattern, linkers were disassembled by TMSD, eventually driving the phase separation of orthogonal droplets (Fig. 2e). The authors reported a 2-layer circuit that recognised a 4-miRNA signature. It is worth noting that the reaction of miRNAs with the self-assembled enzymatic network or the DNA droplet is stoichiometric, which limits their sensitivity. As suggested by the authors, introducing an amplification stage would certainly enhance the overall performance for sensitive detection.

### DNA-based neural networks

Although society has been revolutionized by digital electronics that harness binary computation using Boolean gates, this is not necessarily the most appropriate way to compute with molecules. With this in mind, it is important to consider what distinguishes an electronic device from a molecular device. Firstly, an electronic signal demonstrates an identical state (0 or 1), whereas a biomolecule in solution is often complex. The biomolecule is capable of folding in numerous ways and adopting a multitude of configurations. Secondly, biomolecules interact in complex ways that are challenging to model. Transistors produce a sharp and nonlinear response, while the response of biochemical reactions is more graded and nonlinear. Thirdly, modern lithography enables the spatial wiring of millions of Boolean gates, but chemically wiring millions of biomolecules is presently a herculean task, since a new “chemical” wire based on a specific molecular interaction would need to be created for each connection between two species (*i.e.* linking the output of a reaction to the input of another one). Digital computing allows for computations with arbitrary precision, although it requires extra machinery to suppress low signals and amplify strong ones. This may not be necessary for DNA computing, which is aimed at low-precision computations like detecting the presence or absence of a set of DNA strands. For these reasons, molecular-based binary computation has not enjoyed the same exponential scaling (Moore's law) as the electronics industry: the state-of-the-art in Boolean DNA computing plateaued at 5–10 logic gates for a decade, although some increases have been noted recently.^56^

**Fig. 3:**
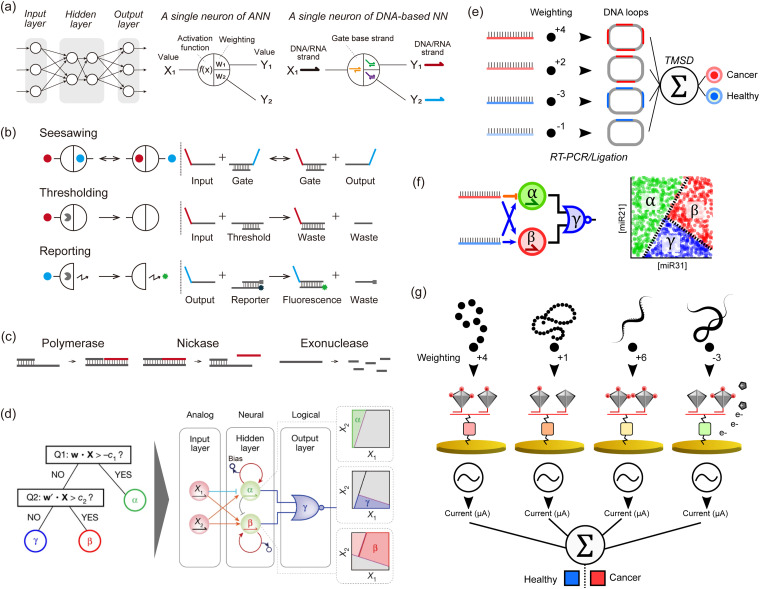
DNA-based neural networks. (a) Schematic model of a neural network and a single neuron of an *in silico*/DNA-based neural network. (b) Schematics of seesawing, thresholding, and reporting reactions, which are the elements of DNA-based neural networks. Solid circles indicate signal strands. Pac-men indicate threshold or reporter complexes. (c) Enzymatic reactions consisting of PEN (polymerase, exonuclease, and nickase) for a DNA toolbox. (d) Illustration of the architecture of neural networks that perform non-linear decision-making (a space partitioning tree). The system involves two strands encoding a position (X1, X2) in the concentration plane, and tests if X is a member of the corresponding half-plane at each of its nodes. The inputs X_1_ and X_2_ are connected *via* 4 converters (3 positive-weighted converters and one negative-weighted converter) to two linear classifiers, producing α and β. The outputs α and β then serve as inputs to a NOR gate, which suppresses a fluorescence of a species γ when either α or β is present. The hidden layer functions as a neural network and decides membership of the α and β region with two linear classifiers. These classifiers are indirectly coupled through competitive inhibition, and membership is read out by fluorescent reporters. The output layer functions logically and decides membership of the γ region with a NOR gate. This gate turns off the fluorescence of the γ region if the position X is a member of either the α or β region. Reproduced from ref. 13 with permission from Springer Nature, copyright 2022. (e) An *in silico*-trained miRNA classifier is encoded in a TMSD reaction cascade, allowing for the identification of lung cancer patients from plasma samples. The input miRNAs undergo a series of biochemical processing steps (RT-PCR and ligation) that result in a DNA loop containing several probe binding sites that correspond to the absolute weight value (red and blue for positive and negative weights, respectively). The DNA loops are used as inputs of a perceptron-like algorithm actuated with TMSD reactions. (f) miRNA-based nonlinear sample classification using a PEN-DNA network encoding a hybrid neuromorphic/Boolean architecture. The miRNA inputs are converted into short oligonucleotides that activate (blue arrow) or repress (orange stroke) the exponential amplification of α, β and γ DNA strands. This system was operated in microdroplets, and the plots show the fluorescence of the α, β or γ reporter in the droplets. (g) Multimodal molecular classifier for prostate cancer diagnosis. Small molecules (red), proteins (orange), miRNAs (yellow), or mRNAs (green) are captured on target-specific gold electrodes and tagged with an HRP-modified DNA tetrahedron, the number of HRP (red disks) per structure defining the weight value. In the presence of the HRP substrate, the electroactive product is detected by cyclic voltammetry, and the signals from each target are analyzed *in silico*.

In response to this stagnation, some groups have departed from the digital approach to mimic more closely the analogue paradigm of computation, especially several types of Artificial NNs (ANNs), which are inspired by neural or genetic processes. ANNs are composed of nodes (‘neurons’) organized into layers that are interconnected by edges (‘synapses’). The input layer receives signals that are processed and transmitted to the output layer, optionally passing through intermediate hidden layers. Each edge has a weight that defines the strength and sign of the connection between two neurons. After summing its inputs, a neuron applies a nonlinear transformation through an activation function, and the output is transferred to the next neurons (Fig. 3a). Indeed, gene regulation networks (GRNs) that compute inside cells, and the electrical neuronal networks that compute in our brains share intriguing similarities that set them apart from digital computations performed in a CPU. Firstly, GRNs and neuronal networks are slow, but they compensate for their high latency with a massively distributed, recurrent, and parallel architecture. Computation is not orchestrated by a single central unit. Rather a global computation emerges from the computations of a myriad of small computation units. Secondly, GRNs and neuronal networks are analogue: they weigh, sum, and compare signals from their inputs. In the brain, neurons are often modelled with a fire-and-integrate model, which prescribes that a neuron fire an output once the sum of its incoming inputs exceeds a threshold. GRNs feature similar architecture, with bow-tie or hourglass motifs, where the expression of a set of proteins is regulated in a shared and non-linear way by various input signals. Thirdly, these types of NNs do not clearly separate computation and memory, but intertwine them in a subtle manner. In the brain, memories are stored in neuronal circuits, which also compute this data while integrating stimuli from the environment. Likewise in GRNs, genomic information is stored in DNA, but DNA also serves as a computing substrate for a myriad of proteins and enzymes (transcription factors, polymerases, *etc.*).

To construct analogue biomimetic circuits, in 2011, Qian *et al.* reported DNA-based NNs powered by cascades of TMSD reactions with DNA strands as inputs (Fig. 3b).^10^ The authors computed iconic Boolean primitives such as XOR, and demonstrated signature features of NNs such as an associative memory on 4 bits, which allows retrieval of information based on partial input cues. This paper convincingly showcased the power of neuromorphic approaches, as similar computations using only Boolean gates would have needed much larger circuits. Following this ground-breaking demonstration with wet-lab experiments, a proposal from 2013^57^ led to the group of Lulu Qian reporting in 2018 a DNA-based NN that recognised handwritten digits encoded in DNA using a combination of seesawing, thresholding, and DNA-reporting reactions (Fig. 3b).^11^ In 2022, the group of Hao Pei reported DNA convolutional NNs that classify pictograms encoded in DNA strands.^12^ Inspired by the convolutional architecture of *in silico* NNs used for computer vision, the authors demonstrated the classification of several dozens of pictograms, each encoded in hundreds of DNA strands. Those DNA-based NNs were mainly based on DNA strands, which were used as the input, signal, and computing substrate. Enzymes were occasionally employed to boost the initial concentrations of inputs but were not part of the implicit operation of the NN. In principle, these DNA-only networks are simple to design, model, synthesise, and debug since the thermodynamics of DNA base-pairing can be reliably computed from first principles. However in practice, kinetic effects that are difficult to model (such as leaks or kinetic traps) represent a major source of flaws, which limits the performance and scaling of those DNA-only networks.

In a parallel stream of investigation, researchers explored the use of enzymes to boost the speed, sensitivity, specificity, or nonlinearity of DNA-based reactions. In a pioneering work in 1999, Hagiya and Nishikawa proposed whiplash PCR, a mechanism for computation that repeats cycles of binding, elongation, and unbinding of the 3′ end of a strand to itself (Fig. 2c).^58^ Whiplash PCR enables autonomous molecular computation by self-directed recursive polymerase extension of a DNA hairpin mixture. In 2008, Takinoue *et al.* reported experimental computation with RTRACS (Reverse-transcription-and-TRanscription-based Autonomous Computing System), which used forward and reverse transcription to compute with DNA and RNA.^59^ In 2018, Kishi *et al.* proposed an isothermal cousin of whiplash PCR, the primer exchange reaction. In this elegant scheme, a primer is consecutively extended by a set of hairpins.^60^ The sequence of elongations is encoded in the sequences of the hairpins, which the authors put to use in computing Boolean primitives. Utilising the aforementioned enzymatic techniques, Kim *et al.* earlier proposed in 2004 one of the first neuromorphic architectures with DNA and enzymes.^61^ Their computational primitive is a transcription gate, which contains a DNA template, an RNA polymerase, and an ssDNA input. The promoter region of the DNA template is single-stranded, which prevents its recognition by RNA polymerase and subsequent transcription. But when an activator input strand is added, it binds to the promoter and turns it into a competent double-stranded promoter – in turn activating transcription by the RNA polymerase (of RNA strands located downstream of the promoter). Building on this motif, the authors simulated transcriptional networks that implemented associative memories – a signature of neuronal systems. Rather than fighting enzymatic saturation, they suggested it could be utilized in a winner-take-all system in which several catalysts are competing. In 2013, it was shown with theory and simulation that a winner-take-all architecture, inspired by sensory networks in the brain, could be more robust and powerful than existing architectures.^57^

In 2011, the group of Yannick Rondelez reported the Polymerase Exonuclease Nickase DNA toolbox (PEN DNA toolbox), a general framework to compute with DNA and enzymes (Fig. 3c).^62^ The toolbox comprises three enzymes: a polymerase, a nickase, and an exonuclease. The polymerase and nickase produce an output strand B, in response to the binding of an input strand A to a template of the form A → B. The exonuclease degrades the ssDNA, which not only maintains the system out-of-equilibrium but also contributes to enforcing subtle dynamics such as bistability. By wiring the templates together, various dynamics such as multistability or oscillation can be implemented. Using this PEN DNA toolbox, Okumura *et al.* reported in 2022 on enzymatic NNs that perform non-linear decision-making (Fig. 3d).^13^ Of note, this was one of the first papers to extensively use droplet microfluidics to optimise and compute with DNA. Microfluidics is capable of miniaturizing chemical reactors, allowing tens of thousands of experimental conditions to be tested simultaneously. First, the balance of enzymatic activity in networks must be carefully tuned, requiring subtle adjustments in enzyme concentrations and incubation temperatures. The authors used massive microfluidic titration and thermal gradients to prepare circuits with various compositions and incubate them in a range of temperatures – rapidly pinpointing the optimal conditions. Secondly, the massive throughput of microfluidics helped in visualizing the behaviour of the neural networks over the input space. Since the inputs of DNA neurons span a range of concentrations (rather than binary values as in Boolean gates), experimentally testing the neurons requires preparing a large number of combinations of inputs at different concentrations. By combining enzymatic networks and microfluidics, Okumura *et al.* demonstrated two key advances. First, they built a linear classifier with performance superior to DNA-only networks. The enzymatic classifier senses input RNA or DNA strands in the picomolar range (as opposed to the nanomolar range for DNA-only networks), and can sense relative variations of 10–20% in the concentrations of the inputs (as opposed to ∼60% for DNA-only networks). This sensitivity to relative concentration changes is crucial for a classifier, as it determines the margin of classification, which is the minimal distance by which input of two distinct classes can be separated. Okumura *et al.* used this improved margin of classification to implement majority voting on 10 Boolean inputs. They leveraged the flexibility of this framework, to give veto or dictator rights to specific strands. Second, they combined neural and Boolean computations to demonstrate a quintessentially non-linear computation: the partitioning of a concentration plane into three regions.

### DNA-based neural networks: towards diagnostics

#### Neuromorphic classifiers

NN architectures, renowned for their capacity to capture intricate patterns (in miRNA concentrations) through activation functions and hidden layers, are seen to outperform Boolean circuits, which are themselves inherently tied to binary input values. When considering chemical reaction networks operating purely in solution, neuromorphic architecture possesses the additional advantage of compactness, which can be harnessed in scaling up circuits, to integrate from dozens to hundreds of miRNA inputs.^11^ Furthermore, these architectures exhibit robustness against noise and adaptability to other classification tasks by adjusting the weights or the neural connectedness.

Zhang *et al.* conducted *in silico* training of a lung cancer classifier based on 4 miRNAs, which was then translated into a TMSD-based neuromorphic algorithm.^63^ The endogenous miRNA targets were amplified by PCR and then converted into DNA loops integrating a number of probe binding sites that correspond to the weight (Fig. 3e). The resulting DNA loops were mixed with a set of probes and reporters to perform weight multiplication, summation and a final binary classification output (diseased or healthy). Okumura *et al.* resorted to the PEN-DNA toolbox to rationally conceive NN-like molecular classifiers endowed with weight modulation, summation, and nonlinear activation functions.^13^ They demonstrated multilayer architectures for the nonlinear classification of one and two-dimensional miRNA concentration space (Fig. 3f). Yin *et al.* employed programmable atom-like nanostructures for the multimodal classification of prostate cancer patients.^64^ The technology involves an array of electrodes functionalised with a myriad of recognition elements such as ssDNAs, antibodies, or aptamers for miRNA, proteins, and small molecules (Fig. 3g). Target molecules are captured on the electrodes and tagged with DNA tetrahedrons whose vertices are modified with HRP. The number of HRP attached to each tetrahedron can be controlled, offering a general strategy for tuning the weight assigned to each target class. After forming sandwiches between the immobilised recognition element, target, and HRP-functionalised tetrahedrons, the electrochemical signal, proportional to the target concentration and its associated weight, is measured in the presence of tetramethylbenzidin as a substrate for HRP. The final classification is however performed *in silico via* a mathematical function, resulting in a hybrid *in moleculo*/*in silico* single-layer perceptron.

## 3. DNA computing with non-nucleic acid inputs

Despite the impressive achievements of the field of DNA-based molecular programming in terms of performing Boolean or analogue computation within molecular media, the type of information that these systems can process is still very limited. The approach has so far been mostly restricted to manipulating information presented as nucleic acid inputs, while computing other molecular inputs is much less common. This is due to a variety of reasons including (i) it is generally straightforward to connect a DNA/RNA-based molecular network to nucleic acid inputs, as in many cases one can simply use the same strategies as those used for the internal nodes or gates of the network; however, connection to other molecular signals requires the creation of dedicated interfacing modules; (ii) molecular networks, even when restricted to processing nucleic acid inputs, are already of great practical importance, for example in molecular diagnostics or as organisers (master clock) for bottom-up nanotech processes.^65^ Indeed, nucleic acid inputs can encode a variety of signals at the molecular level, for example a Boolean string can be directly re-encoded to a 4-letter DNA alphabet, a virtually infinite dictionary of DNA code-words^66^ can be created to represent categorical data, and concentrations of specific strands can encode additional continuous variables, *etc.*

However, looking at biological circuits – arguably the greatest source of inspiration for molecular-level information processing – one notices that inputs often comprise a variety of non-nucleic chemical signals, for example, small molecules. This is likely a consequence of the central importance of small-molecule metabolism for living organisms, but is also dictated by specific advantageous features of these chemicals in the context of information processing, such as an improved diffusion rate (useful in sender-receiver interfaces), a lower synthesis cost, and duality of function: a given metabolite can play both its primary role (*e.g.* as a source of energy) and secondary roles such as inducing the expression of the enzymes necessary for the corresponding process. Examples of small-molecule sensing in natural genetic circuits include the classical *lac* operon, which responds to glucose and allolactose and accordingly adjusts bacterial metabolism,^67^ the various lactones used in bacterial communication,^68–71^ the sensing of chemical parameters such as pH^72^ and metal ion concentrations,^73^ or physical parameters such as light^74^ or mechanical compression.^75^

In many cases, these biological circuits sense small-molecule inputs *via* a mechanism called allostery, *i.e.* protein or enzyme modulation by a ligand interacting at a remote site (with respect to functional residues). This notion was popularised in the early days of systems biology when it was discovered that allolactose could bind a regulatory protein involved in the sugar response, and change its affinity for a particular locus of the bacterial genome.^76^ In the following sections, we explore the sensing of non-nucleic signals in artificial DNA-based molecular circuits, with the goal of extending the range of inputs they can process.

### Networks based on allosteric transcription factors

Allosteric transcription factors (aTFs) are important tools in the field of synthetic biology, which has carefully collected large sets^77–79^ of orthogonal aTFs that can be used for *in vivo* circuit construction.^80^ These circuits can be modular, elaborate,^81^ and have practical applications, for example in toxic metal detection.^82^ Although whole-cell sensors involving allosterically controlled genetic regulation can be cumbersome as live bacterial cells are required for the assay, their architecture can generally be adapted to a cell-free transcription-translation format.^83,84^ In addition, although the set of molecules that can be sensed by natural aTF is necessarily limited, it has been shown that these transducing proteins possess a surprising degree of plasticity and can evolve toward new specificities.^78,85–89^

**Fig. 4:**
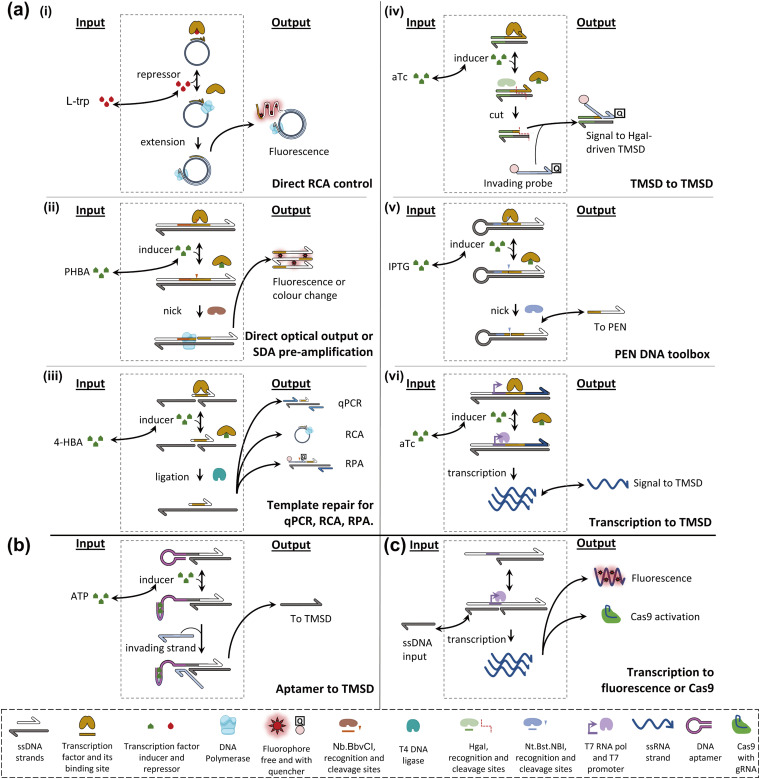
Schematic examples of strategies for DNA computing with non-nucleic acid inputs. (a) aTF-based strategies; (i) TrpR binds the primer-template junction in the presence of l-trp but does not bind in its absence. Phi29 DNA polymerase binds and extends, leading to a fluorescent signal. (ii) HosA transcription factor blocks the Nb.BbvCI binding site unless PHBA is present. In such cases, Nb.BbvCI can nick, leading to nicking-extension cycles producing DNA strands, and optically detectable G-quadruplex formation. (iii) HosA transcription factor blocks a nicked template strand, preventing T4 DNA ligase repair unless 4-HBA is present. In such a case, repair occurs. Subsequently, the repaired strand can serve as a long template in RT-qPCR, RCA, and RPA. (iv) Tetracycline repressor (TetR) blocks the HgaI recognition site. Upon aTc addition, its unbinding allows HgaI to cut, releasing a small ssDNA and exposing the remainder of the complex to TMSD and subsequent cutting cycles. (v) Lactose inhibitor (LacI) blocks the Nt.BstNBI recognition site until IPTG is added. It unbinds and cycles of nicking-extension ensue, producing downstream signals. (vi) TetR blocks the T7 promoter. Upon aTc addition transcription occurs, leading to the RNA oligo downstream signal. (b) Allosteric ribozyme-based strategy. An aptamer sequence is initially blocking the toehold. When ATP is added, the aptamer folds, exposing the toehold to strand invasion and TMSD. (c) A potential aptamer-mediated Cas9-based strategy. A partially double-stranded T7 promoter is completed by an ssDNA input. Transcription occurs, producing RNA that can either bind a fluorophore or activate Cas9 specifically. Such aptamer control of Cas9 has not been demonstrated.

Early attempts to install small-molecule transducers in DNA-based circuits *in vitro* proposed combining an aTF, targeting a species of interest, with an isothermal amplification scheme to achieve sensitive detection of the former. For example, Zhao *et al.* designed a primer containing a tryptophan repressor (TrpR) and complementary to a single-stranded circular template.^90^ In the presence of TrpR and a strand-displacing polymerase, RCA happens in the absence of l-trp, where TrpR is essentially unable to bind DNA, but fails when l-trp is present because TrpR outcompetes the polymerase on dsDNA (Fig. 4a(i)). Based on the level of RCA inhibition, a calibration curve can be constructed (0.5–8 μM dynamic range, LOD – 0.77 μM). The detection of l-trp is specific, as expected from a transcription factor: other amino acids, even close l-trp analogues, do not show a notable effect on the circuit. The group of Weishan Wang later demonstrated a similar approach, where a *p*-hydroxybenzoic acid (PHBA)-specific aTF would block an extension site in a SDA reaction (Fig. 4a(ii)).^91^ PHBA is an industrial antiseptic used in foods and cosmetics, and is also an environmental pollutant. Going one step further in circuit integration, the group added a cross-triggered amplification motif, improving the LOD by a factor of 10, down to nanomolar levels. The approach is also modular since the replacement of the aTF pair with HucR and its cognate binding site enables the detection of uric acid (UA), a disease marker.

The concept of interfacing aTFs with DNA amplification was generalised further by Shanshan Li and Lixin Zhang.^92^ The aTF, now blocking a nicked template site on a primer–template junction, was shown to transduce concentrations of PHBA, UA, and tetracycline, an antibiotic anhydrotetracycline (aTc), to real-time quantitative polymerase chain reaction (RT-qPCR), RCA and recombinase polymerase amplification (RPA). This is in all cases due to competition with a T4 ligase and the successful reparation of the nick which is critical to initiate any of the above-mentioned reactions (Fig. 4a(iii)). Importantly, the sensors worked with river water, human serum, and milk spiked with their potential contaminants. Rodrígues-Serrano and Hsing have adapted the approach to an amplification system based on DNA restriction and TMSD, where aTc (250 nM) and erythromycin (1.25 μM) could be detected specifically when spiked into water samples (Fig. 4a(iv)).^93^ Although these polymerase-free systems may be efficient at signal amplification, this demonstration is also interesting since it has shown that the TMSD mechanism can support a large variety of computational tasks.^94^

Molecular programming toolboxes based on polymerization cycles,^95,96^ such as the PEN DNA architecture,^97^ provide both generic circuit modules and a strong amplification mechanism, enabling ultrasensitive pattern recognition.^13,98^ In addition, these out-of-equilibrium systems can dynamically react to external stimuli, for example, in a push-push memory scenario.^99^ A two-input analogue adder sensing the amino acid tryptophan *via* an aTF has been implemented in the context of PEN,^32^ demonstrating modularity *via* separation of the sensor and processor layers (Fig. 4a(v)). The sensing has even been extended to *in situ* enzymatic activity detection and has been proposed for use in directed evolution *via in situ* primer generation and subsequent PCR in a compartmentalised setting.^100^ Alternatively, the sensing could be combined with previously reported DNA/PEN-controlled circuits,^101,102^ allowing for metabolic feedback loop mimicry.

Julius Lucks combined aTF-controlled T7 RNA transcription with an advanced non-enzymatic computational layer.^103^ Nucleic acid logic gates based on the TMSD mechanism receive RNA inputs whose *in situ* transcription competes with aTF binding, thereby transducing the presence of the small molecule input (zinc and aTc) (Fig. 4a(vi)). Thanks to the modular processing layer, an impressive range of functions was demonstrated: NOT, OR, AND, IMPLY, NOR, NIMPLY, NAND, and an analogue-to-digital converter. The authors suggest that a tunable analogue-to-digital converter can be used for semi-quantitative analysis in the field. They also provide design rules to make aTFs and T7 RNA polymerase (RNAP)-driven transcription compatible with TMSD of DNA. They report that the use of different aTFs is not exactly “plug and play”. This is because the design leads to transcription of the binding sequence of the aTF, which can affect the signal downstream through the formation of unwanted secondary structures.

### Networks based on allosteric nucleic acids

Despite the high affinity of the aTFs for their small molecule ligands (*K*_d_ typically around 10–100s of μM), for their operator sites (*K*_d_ in the region of 1–100s of nM) and the large natural repertoire to choose from, aTFs also have some limitations. First, in the case of *in vitro* applications, the proteins have to be expressed and purified. Their stability is less than that of lyophilised oligonucleotides and, where one would wish to explore a new aTF, the time and cost of obtaining and cloning the corresponding coding sequence (CDS) may be higher than simply ordering custom oligonucleotides. Besides, using a protein as a sensing module in a DNA-based network could be considered to be indirect.

Nucleic acid aptamers therefore provide an alluring alternative. Typically consisting of RNA, these short oligonucleotides capable of binding, and sometimes catalytically responding (aptazymes), to a given molecule offer a good interface between the small molecule world and DNA-encoded systems.^104^*In vivo*, aptamer-based riboswitches^105^ are a key tool for natural genetic circuits (Fig. 2c). Hence, they have also been extensively explored as a way to provide allosteric control of transcription or translation in protein expression.^106–108^ Furthermore, the systematic evolution of ligands by exponential enrichment (SELEX) is well-established for mining functional aptamers with desired specificities from randomized libraries.^109–111^ Aptamers with high-affinity constants have been reported (*K*_d_ single μMs^112^ to single nMs^113^) and used as sensors,^114^ whilst the understanding of general design rules is still expanding.^115,116^ It is also possible to combine multiple aptameric sensing elements into a single *cis* design. For example, Yokobayashi *et al.* designed a theophylline and thiamine pyrophosphate (TPP) riboswitch.^117^ The partially random construct was optimised by genetic selection for improved AND and NAND responses.

The group of Michael Jewett tested the possibility of using an engineered riboswitch architecture for *in vitro* transcription-translation (IVTT) systems with biosensing applications. They tested various reported dopamine aptamers, which were inserted between a promoter and a rho terminator, upstream of the green fluorescence protein (GFP) coding sequence. As expected, dopamine binding modulated the efficiency of termination, and hence the GFP signal. Although the fold change was modest even in the best cases, the *cis* design, fully encoded on a single genetic sequence, could be packaged into an assay for direct detection of dopamine in human urine.^118^ Also in the IVTT context, Greef *et al.* showed that DNA-controlled riboswitches could be integrated into a neural architecture, with molecular neurons performing weighted sum and thresholded activation. In principle, this work, and similar approaches, could be expanded to other riboswitch-compatible inputs.^119^ Although strand-cleaving ribozymes and their DNA analogues have been extensively explored as catalytic tools for molecular circuitry,^4^ few approaches have tested other aptamer functions in simplified (non-translational) circuits. Macdonald *et al.* recently reported a hybrid between a theophylline aptamer and a cleaving DNAzyme, which could be used to transduce the presence of theophylline to DNA-based logic.^120^ Fabry-Wood *et al.* have reported steroid aptamers of medical relevance (for deoxycorticosterone and cortisol) that were compartmentalised inside giant unilamellar vesicles.^121^ To simulate real-world application the vesicles were shown to offer protection of the DNA computing elements from the potentially harsh environment (*e.g.* serum samples) whilst letting steroids diffuse through. Additionally, such compartmentalisation paves the way to downstream component re-use while preventing cross-talk, which may prove to be a powerful tool.^122^ Finally, Zhu *et al.* demonstrated adenosine triphosphate (ATP) transduction to the DNA level (Fig. 4b). Interestingly, they point out that aptamer-based sensing circuits can usually accept both the aptamer ligand and aptamer complement. It could thus be argued that any such circuit contains an OR gate, which is typically followed by an AND gate (provided by the invading strand for the TMSD), before conversion of the DNA output into a fluorescence readout.^123^

### Other approaches

In principle, any allosteric enzyme could be leveraged to create an interface between its effector and a molecular circuit, as long as this enzyme's activity is involved in one edge of this circuit. As such, enzymes with a DNA-processing activity represent a natural focus. Some antibiotics are allosteric inhibitors of DNA/RNA polymerases, but this is unlikely to provide a generalizable route for small molecule interfaces.^124–126^ In the search for a more versatile solution, a number of molecular engineering efforts have steered towards installing allosteric regulation into other enzymes. An example, coming from *in vivo* gene editing control, is provided by Cas9-based systems. Recently the groups of David Liu and Tanja Kortemm have shown that Cas9 activity can be controlled through aptamer-based strategies applied to the guide RNAs.^127,128^ In the former case, the hammerhead ribozyme-containing motif blocks the spacer sequence unless the addition of a ligand leads to the removal of the inhibiting sequence. In the latter, the group has found aptamers that are stabilized by the corresponding ligands to activate Cas9. Cas9 molecular programming was pioneered by the Tom de Greef group (Fig. 4c).^129^ Although they have both used fluorophore-binding aptamers for transcription verification during circuit construction, and dynamically controlled Cas9 activity *via* gRNA production, direct control of Cas9 *via* aptamer response to a small molecule remains to be investigated in this setting.

To conclude, various options have been explored in creating the signal conversion modules required to adapt DNA-based molecular programs to non-nucleic and small-molecule inputs. Although efforts have been put into assessing the generalizability of these approaches, it appears unlikely that a single one could suffice. Instead, some molecular ingenuity may be required for each new target, depending on its chemical nature, the molecular-computational task at hand, and specific requirements such as the dynamic range of sensing. However, the design burden may be alleviated by computational or database approaches. For example, Faulon *et al.* proposed expansion of the range of inputs accessible to the aTF option, by mining enzymatic databases for metabolic cascades that can convert the new input into a compound with a reported aTF.^84,130^

## 4. Using nanopores for the electrical decoding of DNA computations

**Fig. 5:**
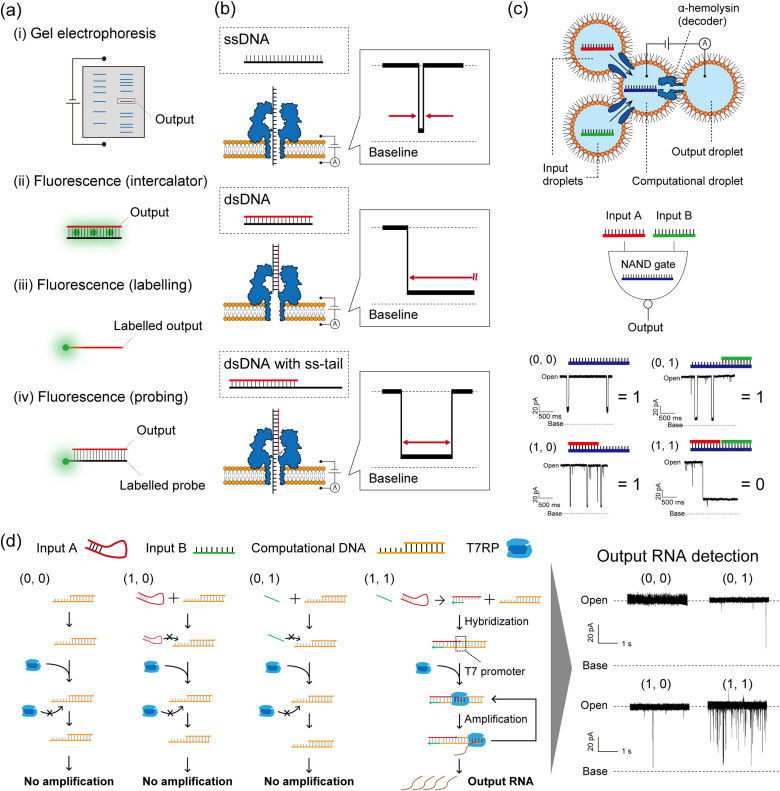
Nanopore decoding for DNA-based logic gates. (a) Conventional decoding for DNA computing. (b) Typical current blocking signals when ssDNA, dsDNA, and dsDNA with an ss-tail are each captured in an αHL nanopore. (c) (top) Schematic illustration of the droplet-based NAND gate with two input droplets, a computational droplet, an output droplet, and αHL nanopores. (bottom) The principle of nanopore decoding for the NAND gate. The input DNAs were designed to bind with the computational DNA, leading to output identification *via* current blocking signals. Reproduced from ref. 131 with permission from PLOS, copyright 2016. (d) (left) Reaction scheme of the AND gate with the transcription system corresponding to each input. (right) Typical current signals resulting from each input pattern. Reproduced from ref. 132 with permission from the American Chemical Society, copyright 2017.

As described in the Introduction section, DNA-computed output information is encoded in nucleic acid molecules, which must then be decoded into a human-recognisable signal. In general, there are several decoding strategies as follows (Fig. 5a):

(i) Detect all the computing products using gel electrophoresis.^1,133^

(ii) Detect the fluorescence of staining reagents in response to the output molecules.^45^

(iii) Detect the fluorescence of dye-labelled output molecules.^134^

(iv) Detect the fluorescence of dye-labelled probes which bind to output molecules.^135^

Method (i) was broadly used in the primary demonstrations of DNA computation such as Adleman's DNA-based parallel computation^1^ and Benenson's DNA-based automaton construction.^133^ Gel electrophoresis is an appropriate method for decoding if the molecular weight or structure of the output DNA molecule differs from those of the other available DNA molecules in the system.^6^ Using such a method in decoding is extremely time-consuming, due to the sequential manipulation of PCR and gel electrophoresis (exceeding several hours). For example, Adleman's method required seven days of laboratory work for decoding. Method (ii) has been proposed in order to skip the gel electrophoresis step, designing an output-responsive amplification reaction followed by an increase in fluorescence intensity derived from staining reagents.^45^ This strategy can be used in conjugation with the output-specific amplification reaction because the dye will stain any double-stranded DNA molecules. Amplification-free approaches have also been adopted in methods (iii) and (iv), using dye-labelled oligonucleotides. In method (iii), fluorescence is directly labelled to output DNA molecules, while the output precursors remain non-fluorescent.^4,9,40,41^ This effect is achieved through the strategic utilization of quenchers and inter- or intramolecular DNA structures. Method (iv) employs dye-labelled DNA probes that fluoresce in response to output DNA molecules. This method can be adapted to amplified output molecules.^136^ In other cases, decoding strategies based on electrochemical^48,64^ or colorimetric detection^5,53,91,92^ have been reported. While the above decoding strategies have been individually employed for specific DNA computations, a simple and universal decoding method would greatly aid in biomedical applications of DNA computation. An example of such a practical approach is the use of nanopore technology in signal decoding.^137^

### Nanopore decoding: principle

Nanopore technology is an effective tool for single-molecule sensing with expanding usage across fields, from detecting DNA,^35^ small molecules,^138^ and glycans^139^ to DNA sequencing,^34^ with great potential for peptide and protein sequencing.^140,141^ In nanopore sensing, a molecule that enters a nanopore due to the application of a voltage is detected as an ionic current blockage, allowing for the electrical discrimination of individual molecules at the single-molecule level. This technique uses pore-forming proteins as nanopores, embedded in a planar lipid bilayer membrane.^142^ One particular nanopore protein, α-hemolysin (αHL) from *Staphylococcus aureus*,^35^ is conventionally used for oligonucleotide detection due to the size compatibility between the pore diameter (∼1.4 nm) and the ssDNA diameter (∼1.0 nm). This size compatibility facilitates the electrical and label-free discrimination of ssDNA and dsDNA (2.0 nm diameter) as follows (Fig. 5b). A ssDNA can pass through the αHL nanopore because its diameter is smaller than that of the pore restriction, resulting in a transient current blocking signal. On the other hand, a dsDNA cannot pass through and clogs the pore due to its larger diameter, resulting in a long current blocking signal. In the case of dsDNA with a single-stranded tail (ss-tail), the clogged double-stranded region can be unzipped and then pass through the pore. This molecular behaviour is reflected as a temporary current blockage followed by a return of the current signal to the pore-opening state. These nucleic acid structures (ssDNA, dsDNA, and dsDNA with an ss-tail), associated with specific current signals, can be programmed as DNA-computed outputs by strategic sequence design. To that end, the strategy of nanopore decoding is to incorporate the output information into the nucleic acid structure by specific sequence design, making it electrically decodable by nanopore sensing from the shape of the current blocking signal. The decoding characteristics of several methods explained above are summarized in Table 1.

**Table 1:** Comparison of the characteristics of different decoding methods

	(i) Gel electrophoresis with fluorescent dyes	In tube			Nanopore
		(ii) Fluorescence (intercalation)	(iii) Fluorescence (labelling)	(iv) Fluorescence (probing)	
Sensitivity to decode output molecules	nM–μM^a^ ^1,3,6^	μM–mM^a^ ^45,90^	nM–μM^9,40^	nM^8,10,44^	pM–nM^b^ ^141,143^
Compatibility with amplification steps before decoding	High	High	n/a^c^	High	High
Decoding time after output molecules produced^d^	Hours	Minutes	Minutes	Minutes	Minutes
Multiplicity of decoding operations in a single equipment	High	Low	Low	Low	Low^e^
Compatibility with *in vivo* computing	n/a^f^	Low	n/a^g^	High	Low
User-friendliness across different labs	High	High	Medium	Medium	Low^h^

a The methods (i) and (ii) are typically conducted after amplification steps. The concentration ranges presented in Table 1 were estimated based on the amplification steps.

b The quantification of output molecules at pM levels, in addition to their decoding, is a time-consuming process that requires several hours.

c Fluorescent molecules are not amplified.

d In the absence of a sufficiently high concentration of output molecules, an amplification step before decoding is required.

e Nanopore methods have a medium multiplicity when GridON (a benchtop device designed to run multiple flow cells of MinION from Oxford Nanopore Technologies) is custom-configured and used as a nanopore decoder. *In vivo*.

f Gel electrophoresis.

g Chemical modification of fluorescent molecules to DNA cannot be performed.

h Nanopore methods are user-friendly when MinION is custom-configured and used as a nanopore decoder.

### Nanopore decoding: demonstration

To demonstrate the nanopore decoding concept, Yasuga *et al.* first constructed a NAND gate using a four-droplet system with αHL nanopore sensing, consisting of two input droplets, one computational droplet, and one output droplet (Fig. 5c).^131^ The αHL nanopore was reconstituted in the interfacial lipid bilayer of the computational/output droplet. The DNAs were designed to form dsDNA only in the case of input (1, 1), otherwise, ssDNA or dsDNA with an ss-tail were formed in the computational droplet. This design principle allowed control of the current signals depending on the input patterns; only input (1, 1) showed a long current blockage (= output ‘0’), otherwise a transient/temporary current blockage was observed (= output ‘1’). In this method, the output molecules in the NAND gate were decoded with nanopore technology, and the entire computation was completed within 10 minutes. Based on this droplet-based nanopore system, two DNA logic gates (OR and NOR) were then cascaded and decoded within 10 min using the nanopore and a movable droplet system.^144^ Ohara *et al.* subsequently attempted to integrate more complex operations involving enzymatic reactions into this droplet-based nanopore sensing system (Fig. 5d).^132^ The designed AND gate consisted of the transcription reaction from DNA to RNA, using two distinct DNA strands as inputs and T7 RNAP as the computational module. Only in the case of input (1, 1), two DNA strands hybridized into the template for the T7 RNAP promoter, resulting in the generation of RNA by transcription. This DNA-computed output was decoded as the event frequency of the RNA-derived transient current blocking signals; the frequency value for the case of output ‘1’ was significantly higher than ‘0’.

In addition to the above basic structures (ssDNA or dsDNA), other nucleic acid structures such as aptamers,^145^ G-quadruplexes,^146^ i-motifs,^147^ and triplexes^148^ can be detected from characteristic current blocking signals by αHL nanopore sensing. Therefore, depending on the sequence design, non-nucleic acid information including small molecules, metal ions, and pH can potentially be used as inputs for DNA computation with nanopore decoding. As an alternative to αHL, MspA^149^ (1.2 nm diameter) from *Mycobacterium smegmatis* was used as the nanopore to decode barcoded information on DNA from its current signals using synthetic nucleic acids,^150^ demonstrating the potential of a wide variety of nanopores as decoders in DNA computation. Besides biological nanopores, solid-state nanopores (*i.e.* glass nanopores) have also been developed to detect and discriminate the DNA structures for decoding the ‘0’ and ‘1’ information encoded in long DNA, with possible applications in the field of DNA storage (for details, please refer to the review papers^137,151^). Following the decoding of binary information as described above, another type of DNA computation, Adleman's parallel computation for solving the HPP, was also decoded using the nanopore sensing technique.^152^ Takiguchi *et al.* solved the small HPP by examining the time of the current blocking signal, which reflects the time of the unzipping phenomenon of dsDNA with an ss-tail. The unzipping time is dependent on the Gibbs free energy of the double-stranded region. A graph with 5 nodes and 10 paths was constructed, and DNAs were designed to produce output molecules that were unzipped through the αHL nanopore. The DNA-computed output information was successfully decoded from the time of the current blockage within a small number of steps compared to Adleman's approach.

### Nanopore decoding: towards diagnostics

Taking advantage of the label-free and electrical sensing capability of nanopores, nanopore decoding has also been applied in diagnostics by directly detecting oligonucleotide biomarkers. Towards diagnostic applications, DNA computing technology can be combined with nanopore decoding in the strategic design of probes that bind to target biomarkers. Several such methodologies for miRNA-targeting DNA computation with nanopore decoding for cancer diagnosis have been reported, from single-channel nanopore setups to the use of MinION for multiplex implementation.

**Fig. 6:**
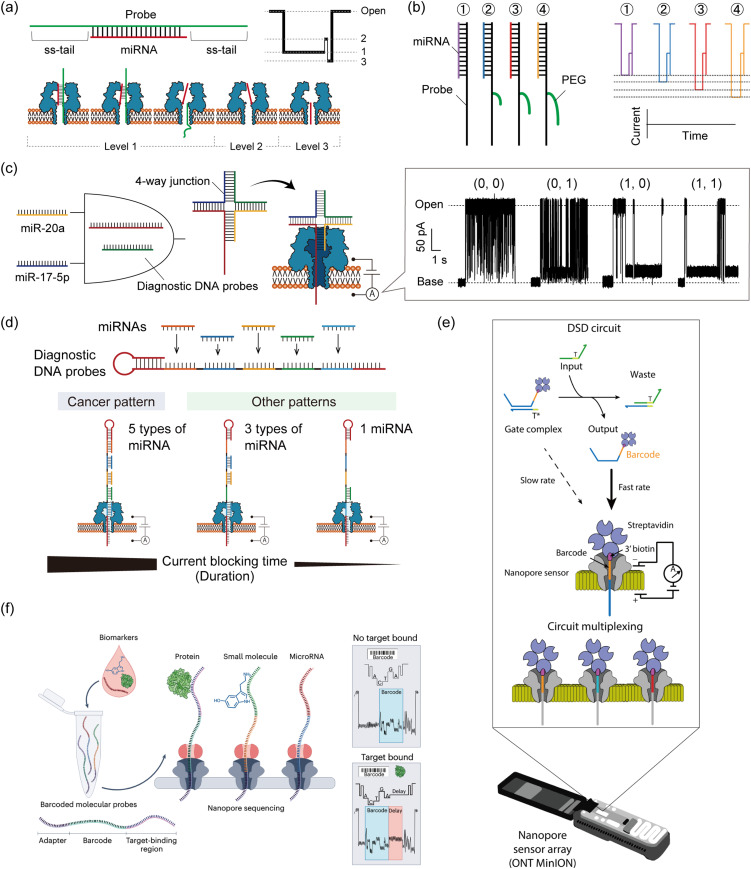
Nanopore decoding for diagnostic applications. (a) (top) The design of the DNA probe to form probe/miRNA duplexes with an ss-tail. (bottom) Characteristic current-blocking signals and respective configurations when probe/miRNA duplexes pass through the αHL nanopore. (b) (left) The design of the PEG-labelled DNA probes (right) and their corresponding current-blocking signals. Reproduced from ref. 154 with permission from the American Chemical Society, copyright 2014. (c) Schematic illustration of the AND gate with nanopore decoding. Diagnostic DNA probes were designed to form 4-way junction structures when two target miRNAs are present simultaneously, resulting in output identification *via* current blocking signals. Reproduced from ref. 155 with permission from the American Chemical Society, copyright 2018. (d) (top) The design of a diagnostic DNA probe that codes the complementary sequences of target five miRNAs. (bottom) Schematic illustration of the principle of nanopore decoding. (e) MinION-based detection of DNA circuit outputs. TMSD-based circuits are mixed and subsequently loaded into the flow cell for real-time readout. Input DNA strands interact with the gate complex, displacing the biotin–streptavidin-labelled output ssDNA which contains a unique barcode situated at the 3′ end of the strand. Once released, this barcode-tagged output ssDNA becomes accessible for capture and analysis by the nanopore sensor. The nanopore sensor can differentiate between various output strand barcodes, enabling the simultaneous monitoring of multiple DNA circuit components. Reproduced from ref. 39 with permission from Springer Nature, copyright 2022. (f) Schematic illustration of nanopore decoding-based diagnostics using MinION devices. The biosample is mixed with a pool of barcoded probes and then subjected to nanopore sequencing. Reads where the probes have bound to their target analyte can be identified by the presence of a delay signal within the raw nanopore-derived current blocking signal. Reproduced from ref. 156 with permission from Springer Nature, copyright 2023.

Prior to the implementation of DNA computation for miRNA diagnostics, several nanopore techniques were proposed for rapid detection. Wang *et al.* reported the detection of miRNA in lung cancer patients using a simple probe design (Fig. 6a).^143^ They designed DNA probes to form probe/miRNA duplexes with an ss-tail, resulting in characteristic current blocking signals when passing through the αHL nanopore. Based on this simple probe design, other studies have explored using specific modifications such as peptide nucleic acid (PNA)^153^ or polyethylene glycol (PEG).^154^ PEG-labelled probes were proposed for detection of multiple miRNAs simultaneously. By labelling different length PEGs to the individual probes that bind to each miRNA species, target-dependent unique current blocking signals could be simultaneously observed with nanopore sensing (Fig. 6b).^154^ In a different approach to multiplex miRNA detection, a logic gate operation for miRNA pattern recognition was proposed. The operation employs miRNAs as inputs and sequence-programmed probes as a computational module.

Hiratani *et al.* developed an AND gate to identify 2 up-regulated miRNAs, miR-20a and miR-17-5p, that are secreted in small-cell lung cancer (Fig. 6c).^155^ They designed diagnostic DNA probes that bind to target miRNAs and form a 4-WJ structure with an ss-tail, which causes a prolonged current blockage in nanopore sensing only when both miRNAs are present simultaneously (= input (1, 1)). By analyzing the time of current blockage (duration), they discriminated four different input patterns: (0, 0), (0, 1), (1, 0), and (1, 1). In a subsequent study, Takeuchi *et al.* developed a strategy to identify the expression patterns of five miRNAs (miR-193, miR-106a, miR-15a, miR-374, and miR-224) up-regulated in bile duct cancer (BDC) (Fig. 6d).^157^ A diagnostic DNA probe, which encoded the complementary sequence of all target miRNAs, was used to bind to 5 miRNAs simultaneously. The duration of the current blocking signal, which was a consequence of the unzipping phenomenon, reflected the miRNA binding information. Using this designed probe and signal duration analysis, they distinguished the miRNA patterns between BDC patients and healthy volunteers using clinical plasma samples. In addition to the up-regulated pattern recognition of miRNAs, Takiguchi *et al.* proposed using the nanopore decoding method for the simultaneous recognition of up-regulated and down-regulated miRNAs (up: miR-19a and down: miR-5100) associated with oral cancer.^158^ Combining the stepwise binding sequence design proposed in Adleman's computation and the nanopore-derived current signal classification, they identified the 4 patterns of miRNA expression involved in up-regulation and down-regulation, even when using clinical serum samples.

Although the LOD of nanopore technology is conventionally around the pM range,^143^ strategies with improved sensitivity are required due to the low abundance of miRNA.^159^ To detect ultra-low concentration biomarkers by nanopore decoding, two main approaches have been developed. One utilizes asymmetric of electrolyte concentrations between the two different droplets. This asymmetric condition generates a water flow and an enlarged electrostatic field. These changes enhance the capture rate of oligonucleotides in the αHL nanopore, resulting in the detection of lower levels of nucleic acids than in the symmetric condition.^143,160^ Another approach is to combine nanopore sensing with an input-responsive oligonucleotide amplification reaction. Based on the isothermal amplification reaction, Hiratani *et al.* constructed a miR-20a-responsive oligonucleotide amplification.^161^ In their system, miR-20a, a biomarker for small-cell lung cancer, activated the reaction by forming a 3-WJ structure with designed DNAs, generating a large number of oligonucleotides with arbitrary sequences. The generated oligonucleotides could be detected by nanopore sensing more frequently than miR-20a itself. By combining the above two approaches, Zheng *et al.* successfully detected 1 fM nucleic acid molecules.^162^

Regarding target specificity, nanopore sensing has been reported to have single-base resolution in experiments with DNA mutations. Using DNA probes that bind complementarily to fully matched targets, single-base mismatched targets have been identified based on differences in the duration of the current blocking signals.^163,164^ Furthermore, Liu *et al.* have identified the type and position of single point mutations by analysing both the current blocking ratio and its duration in αHL nanopore sensing.^165^ miRNAs are also known to have highly homologous family sequences with one or more point mutations, which can be discriminated using nanopore decoding combined with ssDNA,^143^ PNA,^153^ or LNA^166^ (locked nucleic acid) probes with ss-tails.

As described above, various types of DNA computation have been decoded through nanopore sensing. The nucleic acid structure constitutes the output information, which is then decoded from the currentblocking signals. Although the nanopore decoding method could provide a unique approach for rapid, electrical, and label-free decoding, there are two main obstacles to being a universally accessible platform for practical use. (i) Not only the outputs but all the DNA computational components (DNA, RNA, *etc.*) can be detected through nanopores by applied voltage-induced electrophoresis, resulting in off-target-induced current signals that hinder precise decoding. (ii) The experimental setup required is not suitable for non-experts due to the need for specific lab skills.

Regarding (i), machine learning-based current signal classification/identification is emerging as a useful approach to specifically detect the output oligonucleotide in the pool of other varied DNA/RNA components.^167,168^ This approach could enable accurate decoding in the presence of off-target-induced signals. With regard to (ii), the commercialized nanopore array device, MinION,^37^ can bridge the gap between non-experts and nanopore experiments.

### MinION-based nanopore decoding: demonstration

Historically, the nanopore sensing community has been anchored by a small group of academic labs, each using customized single-channel nanopore setups tailored to their specific research endeavors. These custom setups, while pioneering, were primarily confined to niche research domains. However, the recent commercialization of nanopore sensor array devices, especially the introduction of the MinION device by Oxford Nanopore Technologies,^37^ marks a potentially transformative step. While the MinION has predominantly been associated with DNA/RNA sequencing applications, its intended application and potential extend far beyond these. In direct sequencing, the MinION is utilized to read nucleotide sequences, whereas in DNA computing, it is employed to detect specific molecular events and encoded DNA strands. To date, only a handful of studies have ventured into using the MinION for non-standard nanopore sensing experiments,^38,39,169^ that is, non-DNA/RNA sequencing. As the field evolves, the MinION and forthcoming nanopore array platforms are poised to redefine the boundaries of nanopore sensing, ushering in a new era of innovation and broadening the horizons of potential applications. One of these recent studies, as detailed in Zhang *et al.*,^39^ introduced a method to read out the TMSD-based DNA computing reactions using the MinION device (Fig. 6e). This new approach offers a marked departure from traditional reporting methods, such as fluorescent reporters, by utilizing nanopore sensing for the direct and real-time readout of DNA computing reactions. Its enhanced multiplexability allows for the simultaneous detection of numerous unique DNA barcodes, overcoming the limitations of spectral overlap inherent to fluorescent methods. This not only streamlines the detection process but also paves the way for more complex, multiplexed DNA computing experiments. Additionally, the MinION's real-time data acquisition offers the potential for kinetic readouts, providing insights into the dynamics and temporal aspects of DNA computing reactions.

Zhang *et al.* devised a method to detect the single-stranded output released upon activation of the reporter gate. When the output strand is freed, it can be captured by the nanopore, which is otherwise too narrow for the double-stranded gate species to enter. The strand is lodged within the pore using a biotin–streptavidin anchor, producing a sequence-dependent readout. This allows the design of unique barcodes for each output strand, enabling extensive multiplexing. A total of 30 distinct barcodes were developed, achieving high classification accuracy. The concentration-dependent capture rate allows for the quantification and real-time kinetic analysis of TMSD reactions. The MinION's programmable voltage control was used to capture, read, and eject output strands, maximizing data acquisition. The method demonstrated successful 10-way multiplexing in a single experiment.

### MinION-based nanopore decoding: towards diagnostics

MinION-based multiplex decoding was also applied in miRNA-targeting diagnosis. Based on the nanopore decoding of TMSD-based DNA computing reactions (Fig. 6e), Zhang *et al.* simultaneously determined the relative concentrations of multiple let-7 miRNA variants, indicating the method's versatility in diagnostics. The group of Joshua Edel then developed the MinION-based method for highly multiplexed detection of miRNAs using DNA-barcoded probes (Fig. 6f).^156,170^ The barcode sequences produced unique current blocking signals, allowing for a theoretical design space of up to 1.18 × 10^21^ arrangements. The target binding region can be either a complementary sequence (to bind miRNA or DNA) or an aptamer (to bind proteins and small molecules). This method identified targets by decoding barcode sequences and detecting delay events in current signals; the translocation of target-binding probes is decelerated due to size mismatch with the nanopore geometry. Current signals are then classified with or without delay, revealing the presence of an analyte at the single-molecule level. Using this method, the authors detected 40 miRNAs simultaneously even when using clinical serum samples (less than 30 μL), without the need for dye-labelling and amplification. MinION, which is available as a sequencer, can also be used as a powerful decoder by implementing custom settings.

## 5. Conclusions and outlook

DNA computing holds great promise for a wide range of information processing applications, facilitated by the remarkable programmability of the molecular behaviour of artificially designed DNA sequences. Recently, intensive efforts have been directed into biomedical applications, resulting in rapid and substantial progress toward practical implementation over the past decade. In this tutorial review, we have outlined the strategies to construct DNA-based logic gates, circuits, and NNs, to incorporate non-nucleic acid inputs into DNA computation, and to use nanopore technology as a decoding tool, whilst demonstrating miRNA-targeting diagnostic applications. Here, we discuss the potential and challenges of bringing these technologies to practical use in society.

A significant challenge for the practical implementation of DNA computing is its accuracy. The majority of studies are conducted under optimized experimental conditions, whereas real-world samples would require preparatory manipulation as they contain a complex molecular background that can affect the performance of DNA computation. Potential solutions to this challenge include the introduction of functions designed to detect and correct information processing errors in real-time,^171^ as well as the development of systems that decode only specific output molecules.^172^ In addition, in contrast to electronic integrated circuits, whose gates are physically localised and facilitate unidirectional signal transmission, DNA circuits for both nucleic and non-nucleic inputs are diffuse and mixed in an aqueous solution. This represents a major obstacle in the development of integrated molecular circuit devices. To overcome the limitations of DNA strand orthogonality and the difficulty of controlling the random molecular collision, the group of Chunhai Fan proposed the method of using DNA origami as a platform for immobilising and arraying DNA computational components.^44^ The proposed DNA-programmed gate array includes 24 addressable dual-rail gates that can be controlled programmatically, theoretically enabling an astonishing array of over 100 billion different circuits.

TMSD has played a key role in the development of DNA computing, both in advancing the scalability of DNA-based architectures at a socially implementable level and in programming miRNA-induced dynamic molecular behaviour for diagnostics. It is important to note that when using TMSD-based scalable DNA architectures, the TMSD must proceed towards the thermodynamic equilibrium state. This is because TMSD represents the rate-limiting step of the entire computation. In a study by Lulu Qian and Erik Winfree, 130 DNA strands were used to construct a 4-bit square-root circuit with TMSD cascading.^8^ The circuit, consisting of 12 logic gates, required 10 hours to complete the computation, in contrast to 1 second required by silicon-based computers. In order to speed up TMSD for practical use, there are three promising strategies. The first one is the use of strand-displacing DNA polymerase.^46^ Song *et al.* demonstrated the same 4-bit square-root circuit with the use of enzymes, reducing the computation completion time to 40 minutes owing to the accelerated strand displacing capability of the polymerase. Furthermore, the number of DNA strands used in the computation was reduced from 130 to 37. The second strategy is the introduction of mismatches in the strand displacement region, resulting in local thermodynamic instability and acceleration of TMSD.^173,174^ By introducing a mismatch base pair and optimising its position, the group of Andrew J. Tuberfield demonstrated kinetic control of TMSD with tunability across three orders of magnitude. The third approach is the use of polycations such as poly(l-lysine)-*graft*-dextran (PLL-DEX).^175,176^ The group of Atsushi Maruyama proposed the addition of PLL-DEX for charge screening of the DNA. This resulted in a 30-fold acceleration of TMSD, enhancement of nuclease resistance, and robustness against leakage. We believe that continued research on TMSD acceleration will facilitate the development of DNA computing as an alternative or supporting technology to silicon-based computing and rapid diagnostics.

In the realm of miRNA molecular classifiers, one promising avenue of research involves the design of advanced multi-output classifiers. These classifiers are capable of recognizing myriad miRNA signatures, while also incorporating other biomarkers in a multimodal analysis. Such expanded functionality would enable complex diagnosis tests such as pan-cancer screening^177,178^ or patient stratification.^179^ To achieve this, tailored strategies must be devised to convert any marker of interest into universally interpretable DNA strands that can undergo computational tasks. Furthermore, the development of these classifiers must consider the practical limitations of real-world samples, including accessibility and cost-effectiveness. One potential solution is the advancement of simple and cost-effective readout methods, such as nanopore decoding. Taking advantage of DNA computing, these classifiers can seamlessly combine multi-target recognition and signal processing, potentially leading to the creation of low-cost, user-friendly, yet powerful diagnostic tools that could revolutionize healthcare.

Regarding the practical implementation of nanopore decoding, MinION is compact, portable, and easily powered *via* USB, making it accessible and easy to integrate into various setups by simply adding the oligonucleotide-containing electrolyte solution into the flow cell. The ability to acquire data in real time further enhances its appeal, providing immediate insights that were previously difficult to obtain. Leveraging such universally accessible nanopore devices, nanopore decoding provides a powerful tool for harnessing DNA computing in practical applications. This technological bridge has the potential to contribute to the miniaturization of computing devices, one of the pressing issues in this IT era. From the perspective of diagnostic applications, nanopore decoding offers utility in point-of-care testing (POCT) technology due to its high-throughput capability and simplified end-to-end process, again taking advantage of the universal accessibility and portability of the MinION device. One particularly exciting prospect is the potential integration of nanopore arrays with everyday devices like smartphones. By plugging a nanopore device into a smartphone, ubiquitous sensing applications could become a reality.^180^ This integration could revolutionize fields like personal health monitoring, environmental sensing, and more, making advanced molecular sensing a routine part of our daily lives. For example, “molecular apps” could become commonplace, with each app tailored to specific sensing or diagnostic tests.

Leveraging the biocompatibility of DNA molecules, an exciting research direction of DNA computing is the transition from *in vitro* miRNA detection to *in vivo/in cellulo* miRNA sensing: incorporating DNA computing as genetic/logic circuits to use these regulatory molecules in their native environment. This transition presents several challenges such as the integration of a molecular computer into the complex intracellular media, or the delivery of chemical reactants. The group of Yaakov Benenson has been actively exploring the use of synthetic genetic circuits for processing miRNA patterns intracellularly. In a study from 2011, human cell lines were transfected with plasmids encoding a genetic circuit that recognizes a miRNA pattern.^181^ The elementary motif for miRNA sensing is composed of a “double inversion” module that expresses an output protein (*e.g.* a fluorescent protein) when the level of miRNA is sufficiently high, and represses it otherwise. This allows a sharp response of the gene circuit to the target miRNA. Multiple such sensors can be interconnected to create a complete classifier. The authors demonstrated the selective identification of HeLa cervical cancer cells expressing a specific 4-miRNA signature from a mixed-cell population. In a similar approach, Matsuura *et al.* developed a framework for constructing logic circuits that use mRNA gates and RNA-binding regulatory proteins instead of genes and transcription factors.^182^ By using *in vivo* miRNAs as inputs, the mRNA containing different regulatory elements is produced *in vitro* and transfected into mammalian cells to perform Boolean operations. They notably validated a series of logic gates including AND, OR, NAND, NOR, and XOR. Interestingly, the traditional fluorescence output can be replaced by the expression of a pro-apoptotic protein that induces cell death, highlighting the potential of intracellular molecular computation for theranostic applications. The convergence of miRNA detection and classification tasks with a downstream actuation represents an area ripe for exploration, with the selective triggering of cell death demonstrated *in vivo* in several instances.^161,181,183^

In this tutorial review, we highlighted recent advancements in DNA computing and decoding technologies, with a particular focus on nanopore technology. Our hope is for this review to provide useful information for general readers, facilitating a comprehensive understanding of the principles and applications of DNA computing and nanopore decoding, and thereby enabling a diverse range of scientists to further explore and advance these technologies.

## Author contributions

N. T. and R. K. conceptualised the article. S. T. prepared the initial draft of the sections: Introduction, and Conclusions and outlook. S. T., A. J. G., and G. G. prepared the draft of the section: DNA computing *via* logic gates, circuits, neural networks, and their diagnostic applications. V. S. and Y. R. prepared the draft of the section: DNA computing with non-nucleic acid inputs. S. T., N. T. and J. N. prepared the draft of the section: Using nanopores for the electrical decoding of DNA computations. All authors cooperated in the global check of each draft with expert guidance, suggested edits, and supervision from R. K.

## Conflicts of interest

J. N. is a consultant to Oxford Nanopore Technologies.

## Data Availability

No primary research results, software or code have been included and no new data were generated or analyzed as part of this review.
